# Role of Anti-Osteopontin Antibodies in Multiple Sclerosis and Experimental Autoimmune Encephalomyelitis

**DOI:** 10.3389/fimmu.2017.00321

**Published:** 2017-03-23

**Authors:** Nausicaa Clemente, Cristoforo Comi, Davide Raineri, Giuseppe Cappellano, Domizia Vecchio, Elisabetta Orilieri, Casimiro L. Gigliotti, Elena Boggio, Chiara Dianzani, Melissa Sorosina, Filippo Martinelli-Boneschi, Marzia Caldano, Antonio Bertolotto, Luca Ambrogio, Daniele Sblattero, Tiziana Cena, Maurizio Leone, Umberto Dianzani, Annalisa Chiocchetti

**Affiliations:** ^1^Department of Health Sciences, Interdisciplinary Research Center of Autoimmune Diseases (IRCAD), University of Piemonte Orientale (UPO), Novara, Italy; ^2^Department of Translational Medicine, IRCAD, Neurology Unit, University of Piemonte Orientale (UPO), Novara, Italy; ^3^Division for Experimental Pathophysiology and Immunology, Biocenter, Medical University of Innsbruck, Innsbruck, Austria; ^4^Department of Drug Science and Technology, University of Turin, Torino, Italy; ^5^Laboratory of Human Genetics of Neurological Disorders, CNS Inflammatory Unit, Division of Neuroscience, Institute of Experimental Neurology (INSPE), San Raffaele Scientific Institute, Milano, Italy; ^6^Neurology Unit 2, Centro Riferimento Regionale Sclerosi Multipla (CRESM), Azienda Ospedaliero-Universitaria San Luigi, Orbassano, Italy; ^7^ASO Neurologia, Azienda Ospedaliera S. Croce e Carle, Cuneo, Italy; ^8^Department of Life Science, University of Trieste, Trieste, Italy; ^9^Department of Translational Medicine, Medical Statistics Unit, University of Piemonte Orientale (UPO), Novara, Italy; ^10^IRCAD, Neurology Unit, Scientific Institute, Hospital “Casa Sollievo della Sofferenza”, San Giovanni Rotondo, Italy

**Keywords:** osteopontin, multiple sclerosis, autoantibodies, experimental autoimmune encephalomyelitis, vaccination

## Abstract

Osteopontin (OPN) is highly expressed in demyelinating lesions in multiple sclerosis (MS) and experimental autoimmune encephalomyelitis (EAE). OPN is cleaved by thrombin into N- (OPN-N) and C-terminal (OPN-C) fragments with different ligands and functions. In EAE, administering recombinant OPN induces relapses, whereas treatment with anti-OPN antibodies ameliorates the disease. Anti-OPN autoantibodies (autoAbs) are spontaneously produced during EAE but have never been detected in MS. The aim of the study was to evaluate anti-OPN autoAbs in the serum of MS patients, correlate them with disease course, and recapitulate the human findings in EAE. We performed ELISA in the serum of 122 patients collected cross-sectionally, and 50 patients with relapsing–remitting (RR) disease collected at diagnosis and followed longitudinally for 10 years. In the cross-sectional patients, the autoAb levels were higher in the RR patients than in the primary- and secondary-progressive MS and healthy control groups, and they were highest in the initial stages of the disease. In the longitudinal group, the levels at diagnosis directly correlated with the number of relapses during the following 10 years. Moreover, in patients with active disease, who underwent disease-modifying treatments, autoAbs were higher than in untreated patients and were associated with low MS severity score. The autoAb displayed neutralizing activity and mainly recognized OPN-C rather than OPN-N. To confirm the clinical effect of these autoAbs *in vivo*, EAE was induced using myelin oligodendrocyte glycoprotein MOG_35–55_ in C57BL/6 mice pre-vaccinated with ovalbumin (OVA)-linked OPN or OVA alone. We then evaluated the titer of antibodies to OPN, the clinical scores and *in vitro* cytokine secretion by spleen lymphocytes. Vaccination significantly induced antibodies against OPN during EAE, decreased disease severity, and the protective effect was correlated with decreased T cell secretion of interleukin 17 and interferon-γ *ex vivo*. The best effect was obtained with OPN-C, which induced significantly faster and more complete remission than other OPN vaccines. In conclusion, these data suggest that production of anti-OPN autoAbs may favor remission in both MS and EAE. Novel strategies boosting their levels, such as vaccination or passive immunization, may be proposed as a future strategy in personalized MS therapy.

## Introduction

Multiple sclerosis (MS) is an inflammatory disease of the central nervous system (CNS) characterized by an autoimmune attack against the myelin sheaths and axons resulting in demyelination and axonal loss (1). MS patients display variable clinical courses; at onset, approximately 15% of patients display a primary-progressive (PP) form, whereas the remainder start out with a relapsing–remitting (RR) form, and most of those patients switch to a secondary-progressive (SP) form within 10–30 years (2). An increasing number of disease-modifying treatments (DMTs) are available for RR MS, and a key challenge in therapeutic decision-making is effective treatment stratification, given uncertain prognoses (3).

Data obtained in animal models and humans strongly suggest that osteopontin (OPN) plays a role in the pathogenesis of MS (4). OPN is a 60 kDa secreted phosphoprotein functioning as a free cytokine in body fluids or as an immobilized extracellular matrix molecule in mineralized tissue (5). Its expression is increased in the sera of patients with several autoimmune diseases, including MS (6–9), and it may influence the development of autoimmunity through its immunoregulatory and proinflammatory effects. The OPN transcript is abundant in plaques dissected from the brains of MS patients, whereas it is absent in control brain tissue (10). A similar finding has been obtained in experimental autoimmune encephalomyelitis (EAE), an animal model of MS (10, 11). In blood and cerebrospinal fluid of MS patients, OPN levels are increased and correlate with the clinical stage because higher levels have been detected in RR-MS patients than in PP-MS and SP-MS patients (6). Moreover, in RR-MS patients, OPN levels increase during relapses and decrease in the remission phase without substantial influence by interferon (IFN)-β treatment (12).

Genetic analyses have associated variations of the *OPN* gene with MS (6). In this context, we found variants of the *OPN* gene that were associated with (i) increased risk for MS (an approximately 1.5-fold increase); (ii) severe disease course, with fast switching from a RR to a SP form and evolution of disability; and (iii) production of high levels of OPN because of increased stability of the encoded mRNA (6, 13).

In MS lesions, high OPN levels are present in the perivascular cuff, which surrounds the inflamed blood vessels, contains inflammatory lymphocytes, and is delimited by the endothelium and the basement membrane. At this site, OPN may play a role in lymphocyte recruitment into the MS lesion, which involves α4β1 integrin, the target of natalizumab, an established DMT. During inflammation, thrombin acts on a cleavage site located in the middle of the OPN sequence, near to an arginine–glycine–aspartate (RGD) motif involved in binding several integrins (14), to generate two OPN fragments, one N-terminal (OPN-N) and one C-terminal (OPN-C). OPN-C contains the CD44-binding site involved in the downregulation of interleukin (IL)-10 expression and inhibition of lymphocyte apoptosis (15). OPN-N contains the RGD motif and two cryptic α4β1 integrin-binding sites unmasked by thrombin cleavage, and it is involved in the induction of IFN-γ secretion in T cells (14, 16). The functional activity of the two fragments has been mostly distinguished *in vitro* (17), but the observation that, in carotid plaques of patients with hypertension, levels of OPN-N are higher than those of OPN-C suggests that the two fragments may play different roles also in pathologic conditions *in vivo* (16).

Chabas et al. showed that OPN^−/−^ mice were resistant to progressive EAE and had frequent remission; notably, myelin-reactive T cells produce more IL-10 and less IFN-γ in these mice than in their wild-type counterparts (10). Moreover, treatment with OPN exacerbated EAE in both wild-type and, to a greater extent, OPN^−/−^ mice. In OPN^−/−^ mice, daily administration of OPN during the spontaneous recovery of EAE counteracted the ongoing remission and induced a relapse followed by a progressive severe disease, leading to death (18).

We have recently shown that thrombin-mediated cleavage of OPN plays a role in OPN-mediated relapse induction, since a recombinant OPN-mutant resistant to thrombin-mediated cleavage was less effective than wild-type OPN in inducing the EAE relapse, and OPN-C was more effective than OPN-N (17).

Steinman et al. showed that EAE induction triggered the production of anti-OPN autoantibodies (autoAbs), and remission occurred when their titers peaked (19). Moreover, DNA vaccination with a plasmid encoding OPN before EAE induction boosted the production of these autoAbs and ameliorated the chronic course of the disease. In humans, autoAbs against OPN have been reported in rheumatoid arthritis and osteoarthritis, and their serum level was inversely correlated with markers of disease activity (20). Moreover, passive immunization with antibodies against the cryptic epitope of OPN-N exerted beneficial effects in mouse and primate models of rheumatoid arthritis (21). These data are in accordance with reports showing the production of autoAbs against inflammatory cytokines in several autoimmune diseases and suggesting that they may play a role in counteracting the pathological response (22).

The aim of the research reported here was to evaluate anti-OPN autoAbs in the serum of MS patients, to determine their correlation with the disease course, and to perform preclinical studies assessing the possible use of anti-OPN immunization in MS therapy. The results showed that high levels of anti-OPN autoAbs are displayed by RR-MS patients, especially in the remission phase, and may have a prognostic value at diagnosis. These autoAbs displayed neutralizing activity, mainly recognized OPN-C, and decreased disease severity in EAE.

## Materials and Methods

### Patients

We enrolled two groups of MS patients diagnosed according to the 2001 McDonald criteria (23):

i) a cross-sectional group of 122 patients (73 females, 49 males, mean age 43 ± SD 11 years). Seventy-two patients had RR-, 29 PP-, and 21 SP-MS course. Patients were enrolled from the MS Centers of the Maggiore University Hospital (Novara), San Luigi Hospital (Orbassano, Turin), and San Raffaele Hospital (Milano). Patients and controls were Caucasian, Italian, and unrelated. Healthy controls (HCs, *n* = 40) did not differ in age and gender from the patients.
ii) a longitudinal group of 50 bout-onset patients, recruited during a prospective study aimed at identifying predictors of disease progression (24). We included patients still on regular follow-up 10 years after diagnosis, and of whom we stored serum at time of diagnosis. Clinical data collected in the prospective cohort included age at onset, expanded disability status score, number of relapses updated at 10 years, and any DMTs. We also calculated the Multiple Sclerosis Severity Score (MSSS) at 10 years for each patient (25). DMTs included both first line (beta-IFN and glatiramer acetate) and second line treatments (azathioprine, natalizumab, and fingolimod). Clinical features of the longitudinal cohort are shown in Table 1. Patients were enrolled from the MS Centers of the Maggiore University Hospital (Novara) and Santa Croce e Carle Hospital (Cuneo).

**Table 1:** **Clinical variables in multiple sclerosis (MS) patients followed for at least 10 years**.

	MS patients with 10-year follow-up (*n* = 50)
Female, *n* (%)	28 (56)
Follow-up, years, mean (±SD)	12.0 (±1.2)
Age of onset, years, mean (±SD)	32.0 (±9.9)
Osteopontin (OPN) levels, pg/ml, mean (±SD)	4,952 (±3,441)
OPN autoantibody levels, optical density, mean (±SD)	0.4860 (±0.3415)
Mono-symptomatic onset, *n* (%)	24 (48)
Expanded disability status score (EDSS) at first relapse, median (min–max)	2.5 (1–7.5)
EDSS at last visit, median (min–max)	2 (0–9)
Multiple Sclerosis Severity Score, mean (±SD)	2.7 (±2.4)
*N* relapses at 5 years, median (min–max)	3 (1–14)
*N* relapses at 10 years, median (min–max)	5 (1–23)
MS course at 10 years, *n* (%) – Relapsing–remitting – Secondary progressive	45 (90) 5 (10)
Disease-modifying treatment, *n* (%)	32 (64)

Written informed consent was obtained from all subjects, and analyses were conducted according to the Declaration of Helsinki and approved by the ethical committee of University Hospital of Novara (reference no. CE1804).

### Cloning and Production of OPN Recombinant Proteins

cDNA coding for the human and murine OPN full-length (OPN-FL), OPN-N, and OPN-C were cloned by PCR into a pUCOE expression vector as previously described (17) and stably transfected in Chinese hamster ovary cells (CHOs). Cell supernatants were collected, and the recombinant proteins were purified on a nickel-nitrilotriacetic agarose resin (Qiagen, Limburg, Netherlands) and characterized by Western blotting using either an antibody directed against the His tag (Tetra-His Antibody, Qiagen, Valencia, CA, USA) or an anti-OPN antibody directed against an epitope located in the N- or C-terminal half of the molecule: SPP1 polyclonal antibody (Invitrogen) and polyclonal anti-osteopontin antibody (Millipore, Billerica, MA, USA), respectively (17).

### Detection of Anti-OPN AutoAbs by ELISA

Serum anti-OPN autoAbs were assessed by a custom-made immunoenzymatic assay (ELISA). Briefly, polystyrene ELISA Maxi-Sorp plates (Nunc, Roskilde, Denmark) were coated by overnight incubation at 4°C with 2 μg/ml of the FL-, N-, or C-OPN (recombinant OPN) as capture protein. Non-specific binding was blocked by 1 h incubation with 0.3 ml of 0.05% Tween 20 in phosphate buffered saline (PBS), pH 7.4. Each serum sample (0.1 ml diluted 1:100 in 0.05% Tween 20 in PBS, pH 7.4) was added in duplicate and incubated for 2 h at 37°C. After 10 washes with 0.05% Tween 20 in PBS, antibody binding was revealed by peroxidase-conjugated goat anti-human immunoglobulin (Ig)G (dilution 1:4.500) (Dako, Glostrup, Denmark). The results were expressed as optical density at 450 nm.

### Immune Complexes Dissociation

Heat-mediated dissociation of immune complex was obtained by boiling diluted serum samples and each point of the OPN standard curve for 5 min. After 4 min chill on ice, samples were loaded into a 96-well plate for ELISA evaluation, and the results were compared to non-boiled samples. Concentration of OPN was measured by commercial ELISA according to the manufacturers (R&D system, Minneapolis, MN, USA). Absorbance was detected with a microplate reader (Bio-Rad, Hercules, CA, USA), and the I-smart program was used to calculate the standard curve.

### Activation-Induced Cell Death (AICD)

Activation-induced cell death was evaluated on T cell lines obtained by activating PBMC with phytohemagglutinin (PHA) at days 0 (1 μg/ml) and cultured in RPMI 1640 medium + 10% FBS + IL-2 (2 U/ml) (Sigma, Saint Louis, MO, USA) for 6 days. In the AICD assay, cells (5 × 10^4^/well) were cultured in wells coated with anti-CD3 mAb (OKT3, 10 μg/ml) with RPMI + 5% FBS + IL-2 (1 U/ml) in the presence or absence of recombinant OPN (1 μg/ml). AICD was performed also in the presence of IgG purified (10 μg/ml), with protein G sepharose resin (Ge Healthcare, Piscataway, NJ, USA), from three patients displaying high levels of anti-OPN autoAbs. The control was done by using a commercial goat anti-OPN-neutralizing Ab (R&D system). Live cells were then counted in each well using the trypan blue exclusion test. Assays were performed in triplicate and results were expressed as relative cell survival % calculated as follows: (total live cell count in the assay well/total live cell count in the respective control well) × 100.

### Mouse Vaccination and EAE Induction

Mouse OPN-FL, OPN-N, and OPN-C were cross-linked to ovalbumin (OVA) (Sigma) with glutaraldehyde (Sigma) as reported (26).

The experimental protocol and animal handling were approved by the ethical committee of the University of Piemonte Orientale (reference no. 10821, 10/2013), Novara, Italy. Four-week-old female C57BL/6 mice (*n* = 8/10 each group) were anesthetized with isoflurane and immunized weekly intra-peritoneally for 4 weeks with 10 μg of OPN-FL/OVA, 5 μg of OPN-N/OVA or OPN-C/OVA or 10 μg of OVA in 50 μl glycine buffer, 0.15 M, pH 5.7, and 50 μl of incomplete Freund adjuvant (Sigma). EAE was induced 1 week after the last immunization with 200 μg of MOG_35–55_ peptide (Espikem, Florence, Italy) and scored as reported (22).

Mouse splenocytes were purified and cultured in the presence or absence of 10 μg/ml MOG_35–55_ as reported. After 5 days, levels of IFN-γ, IL-10, IL-4, and IL-17 were evaluated by ELISA (Biolegend, San Diego, CA, USA) in the supernatants and cell proliferation by incorporation of [^3^H] thymidine (27).

### Recombinant mAb Production

OPN full-length was used as a target for selecting antibodies from a phage display Ab library as previously described (28). The selection was performed in immuno tubes coated with recombinant protein incubated overnight at 4°C in PBS. The panning procedure was repeated twice. In total, 96 random clones were selected, and a specific positive clone to OPN-C was identified using phage ELISA. The positive scFv clone was converted into the human scFv–Fc format by subcloning into the pMB-SV5 (29) vector containing the human hinge–CH2–CH3 domain. For antibody production, CHOs cell lines were transfected and stable clones obtained through selection with hygromicin B (500 μg/ml, Invitrogen). The scFv–Fc molecules were purified from cell culture supernatant using a HiTrap protein G column (GE Healthcare). After elution, the preparations of purified scFv–Fc were dialyzed in BupHTM phosphate buffer (Thermo Fisher Scientific, Waltham, MA, USA), aliquoted, and stored at −80°C. The selected mAb was tested by ELISA for its capacity to bind both human and mouse OPN-C and OPN-FL. The mAb was also tested during EAE in a passive immunization protocol.

### Statistical Analysis

The non-parametric Mann–Whitney *U*-test was used to compare autoAb levels in the different groups of subjects; the Pearson correlation coefficient was used to test correlations. The Wilcoxon test was used to compare autoAbs to OPN-C and OPN-N in each subject; the Friedman ANOVA test for repeated measures followed by Dunn’s multiple comparison was used to compare the daily clinical EAE score (GraphPad Software, San Diego, CA, USA). *p* Values below 0.05 were considered statistically significant. The statistical analyses were performed with GraphPad Instat software (GraphPad Software).

## Results

### Detection of Anti-OPN AutoAbs in MS Patients

**Figure 1:**
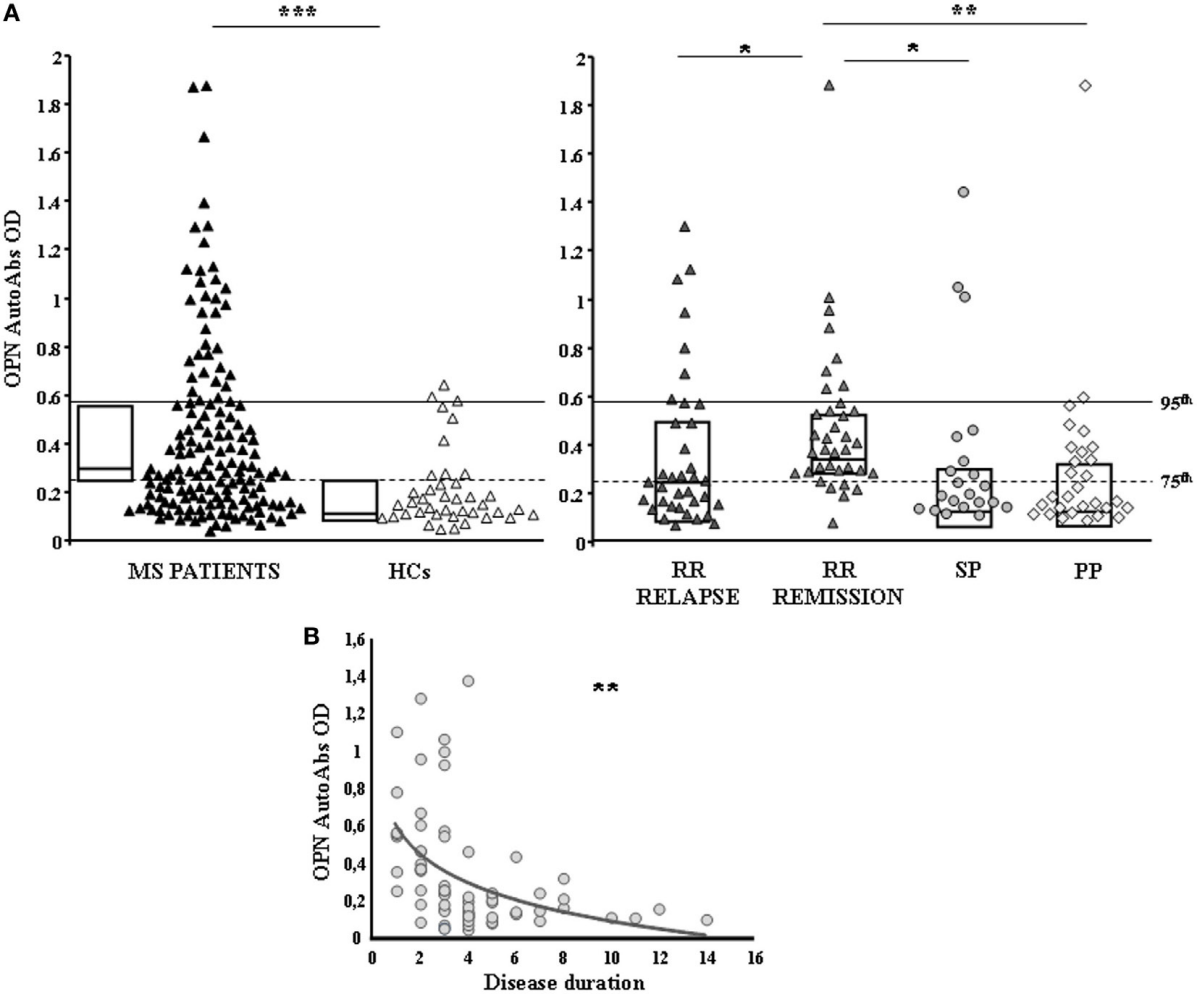
**Anti-OPN autoantibodies (autoAbs) are increased in relapsing–remitting (RR) patients compared with secondary-progressive (SP) and primary-progressive (PP) patients and healthy controls (HCs)**. **(A)** AutoAbs to osteopontin (OPN) in multiple sclerosis (MS) patients and HCs. AutoAbs detected by ELISA using OPN full-length as the capture protein. In the left panel, black triangles mark MS patients and white triangles the HCs. In the right panel, patients are stratified according to their clinical form at the time of blood withdrawal. Dark gray triangles: RR-relapse MS patients; light gray triangles: RR-remission MS patients; gray circles: SP-MS patients; gray diamonds: PP-MS patients; boxes: interquartile range with medians. *p* Values were calculated with the Mann–Whitney *U*-test. The horizontal lines indicate the 75th (dashed line) and 95th (continuous line) percentiles of the HCs. **(B)** Inverse correlation between autoAbs to OPN and disease duration in RR patients (Pearson correlation test) (*p < 0.01, **p < 0.001, ***p < 0.0001).

Serum anti-OPN autoAbs were evaluated in 122 cross-sectional MS patients (72 RR, 29 PP, 21 SP) and 40 HCs by ELISA using OPN-FL. Anti-OPN autoAbs were detected in the serum of both patients and controls, but the titer was significantly higher in patients than in the controls (0.278 vs 0.147, *p* < 0.0001; Figure 1A). Analysis of the different MS clinical forms showed that autoAb levels were significantly higher in RR patients in relapse or remission than in SP and PP patients and HCs (0.289 vs 0.168 vs 0.169 vs 0.147; Figure 1A). By contrast, no differences were detected among SP and PP patients and HCs. Moreover, in RR-MS patients, the autoAb levels were higher in those in remission than in those in relapse (0.368 vs 0.237; *p* < 0.01), and they displayed an inverse correlation with disease duration at the time of blood withdrawal, which was not evident in the other disease courses (Figure 1B). These data suggest that the inflammatory phase of the disease, displaying high levels of OPN, drives production of anti-OPN autoAbs.

### Anti-OPN-FL AutoAbs at Diagnosis and Clinical Outcome

**Figure 2:**
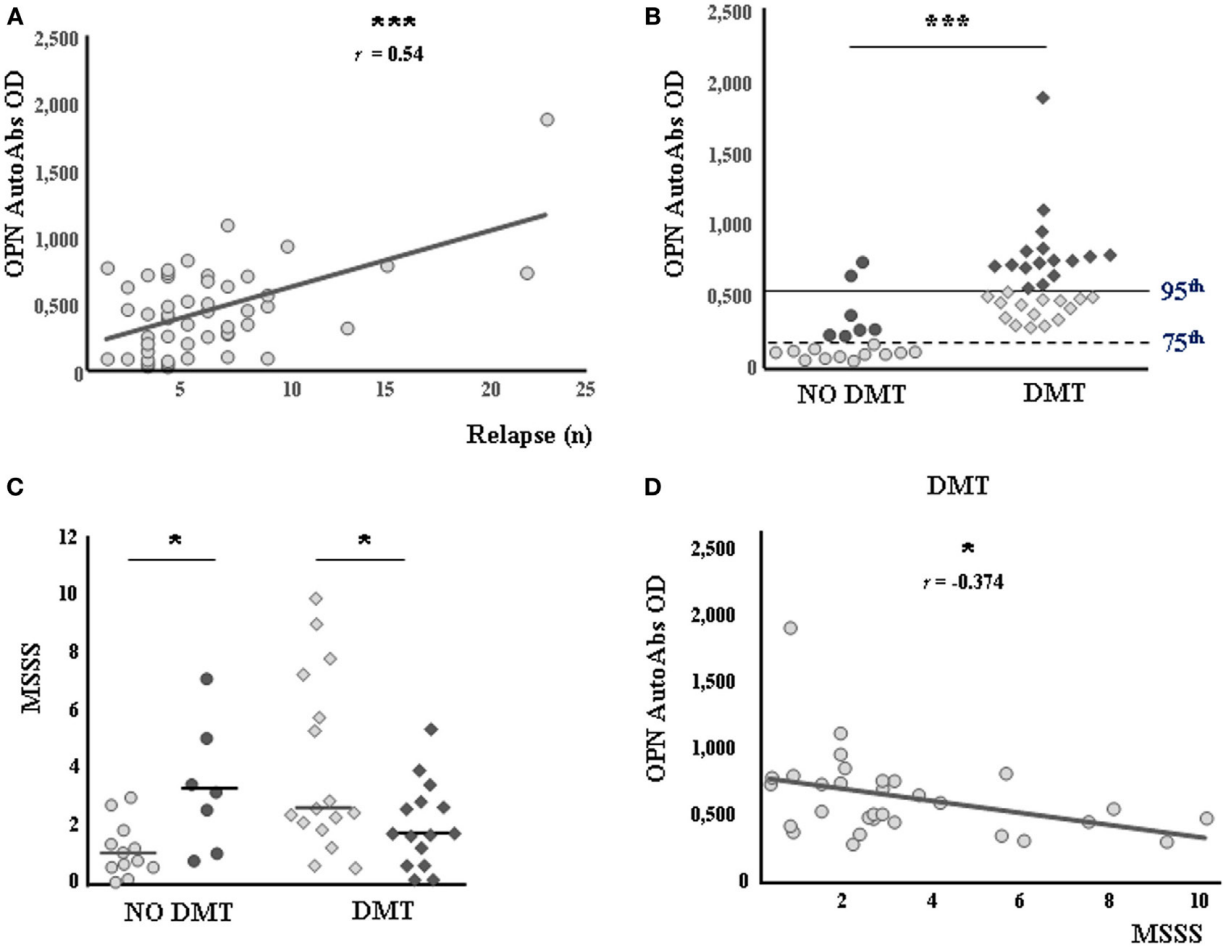
**Autoantibodies (autoAbs) to osteopontin (OPN) at diagnosis predict therapeutic benefits and a reduced Multiple Sclerosis Severity Score (MSSS)**. **(A)** Direct correlation between anti-OPN autoAbs and the number of relapses occurring over 10 years (Pearson correlation test). **(B)** Anti-OPN autoAbs in patients not receiving (circles) or receiving (diamonds) disease-modifying treatments (DMTs). The horizontal lines indicate the 75th (dashed line) and 95th (continuous line) percentiles of the healthy controls. Low expressors and high expressors of each group are shown in pale color and in dark color, respectively. **(C)** MSSS in patients with or without DMTs. Low expressors are shown in pale color; high expressors are shown in dark color, as in the previous panel. **(D)** Negative correlation between autoAbs to OPN and the MSSS in the treated group (Pearson correlation test) (*p < 0.05, ***p < 0.0001).

The second cohort of patients comprised 50 consecutive bout-onset patients followed up for more than 10 years whose serum was withdrawn at diagnosis. In these patients, we evaluated the serum levels of anti-OPN autoAbs and determined their correlation with the clinical outcome after 10 years. The results showed a direct correlation between autoAb levels at diagnosis and the number of relapses occurring in the following 10 years (*r* = 0.542, *p* < 0.0001; Figure 2A). By contrast, no correlation was found with the MSSS after 10 years (*r* = −0.029, *p* = 0.837). Moreover, the autoAb levels were higher in patients who subsequently received DMTs (*n* = 31) than in those who remained untreated (*n* = 19, *p* < 0.0001; Figure 2B). Accordingly, all patients subsequently treated (*n* = 31) displayed levels higher than the 75th percentile of HC values, whereas most untreated patients (*n* = 12/19, 63%) displayed levels lower than this cutoff. Using the 75th percentile of HC values as a cutoff in untreated patients, we found that the MSSS after 10 years was significantly lower in patients with low autoAb levels (*n* = 12) than in those with high levels (*n* = 7) (median 1.01, range 0.64–1.53 vs 3.25, 1.90–4.3, *p* = 0.020; Figure 2C). By contrast, when the cutoff was set at the 95th percentile of HC values in treated patients, the MSSS after 10 years was lower in those with high autoAb levels at diagnosis (*n* = 15) than in those with low levels (*n* = 16) (median 1.70, range 0.96–2.78 vs 2.54, 2.09–6.18, *p* = 0.030; Figure 2C). Consistently, we found that autoAb levels and the MSSS displayed a significant inverse correlation in treated patients (*r* = −0.374, *p* = 0.038; Figure 2D). Finally, no difference in number of relapses at 10 years was found on comparing both treated and untreated patients with different autoAb levels (data not shown).

### *In Vitro* AutoAbs Characterization

**Figure 3:**
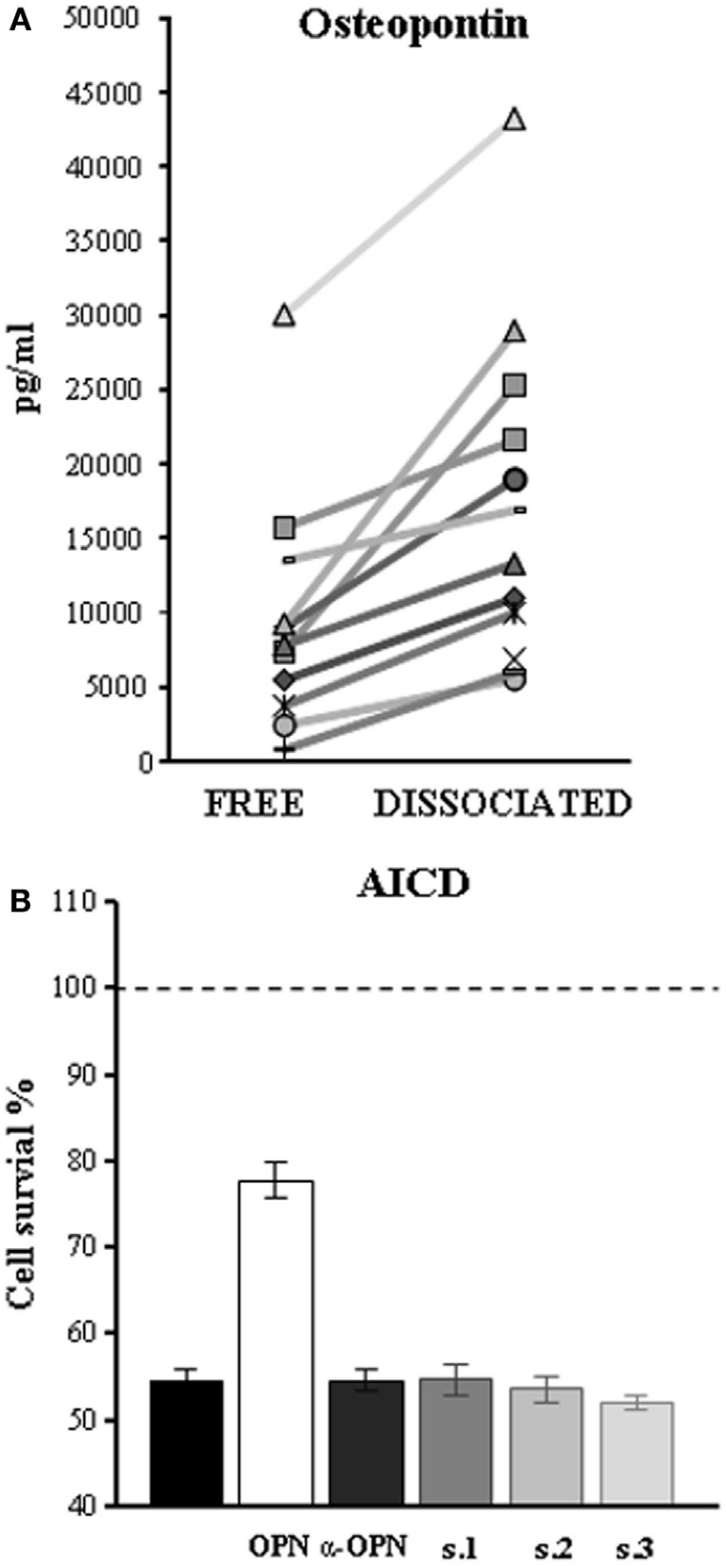
**Autoantibodies (autoAbs) to osteopontin (OPN) in serum of multiple sclerosis (MS) patients form immune complexes and neutralize OPN activities**. **(A)** Heat-mediated dissociation of immune complexes increases the amount of OPN in the sera of MS patients as detected by ELISA. Each symbol represents serum from a different patient. **(B)** AutoAbs to OPN reverse inhibition of activation-induced cell death (AICD) mediated by OPN, by neutralizing its effect. AICD was induced by treating phytohemagglutinin-activated PBMC cell cultures with anti-CD3 mAb (black histogram) and cell survival detected 18 h later; results are expressed as relative cell survival % by setting 100% on the cultures performed in the absence of the anti-CD3 mAb and their means (dashed line). Histograms represent AICD in the presence of recombinant OPN (white), a commercial-neutralizing antibody to OPN (dark gray), or serum of three high-expressor patients (s1, s2, and s3).

Since serum contains both OPN and anti-OPN autoAbs, these may react to form immune complexes *in vivo*, thus blocking the cytokine activity and facilitating its removal from the bloodstream. To assess this possibility, we evaluated the amount of OPN in the sera before and after heat-mediated immune complexes dissociation. The results showed that heat increased the amount of OPN detected in all of the tested sera (Figure 3A). This was not ascribable to unmasking of cryptic epitopes by heat, since boiling did not increase the amount of OPN detected in the standard curve by our ELISA (data not shown).

Since we had previously demonstrated that OPN inhibits AICD (17), we used this test to investigate the autoAbs-neutralizing properties on OPN biological activity. AICD was induced in PHA-activated PBMC from healthy donors in the presence and absence of OPN-FL and each of three preparations of IgG purified from patients displaying high levels of anti-OPN autoAbs (>75th percentile of the controls). Anti-human OPN polyclonal antibodies were used as positive control of OPN-neutralizing antibodies. The results showed that all IgG preparations were able to neutralize the protective effect of OPN-FL on AICD at the same level as the anti-OPN-neutralizing Ab (Figure 3B).

### AutoAbs to OPN-C Are Higher than Those to OPN-N in MS Sera

**Figure 4:**
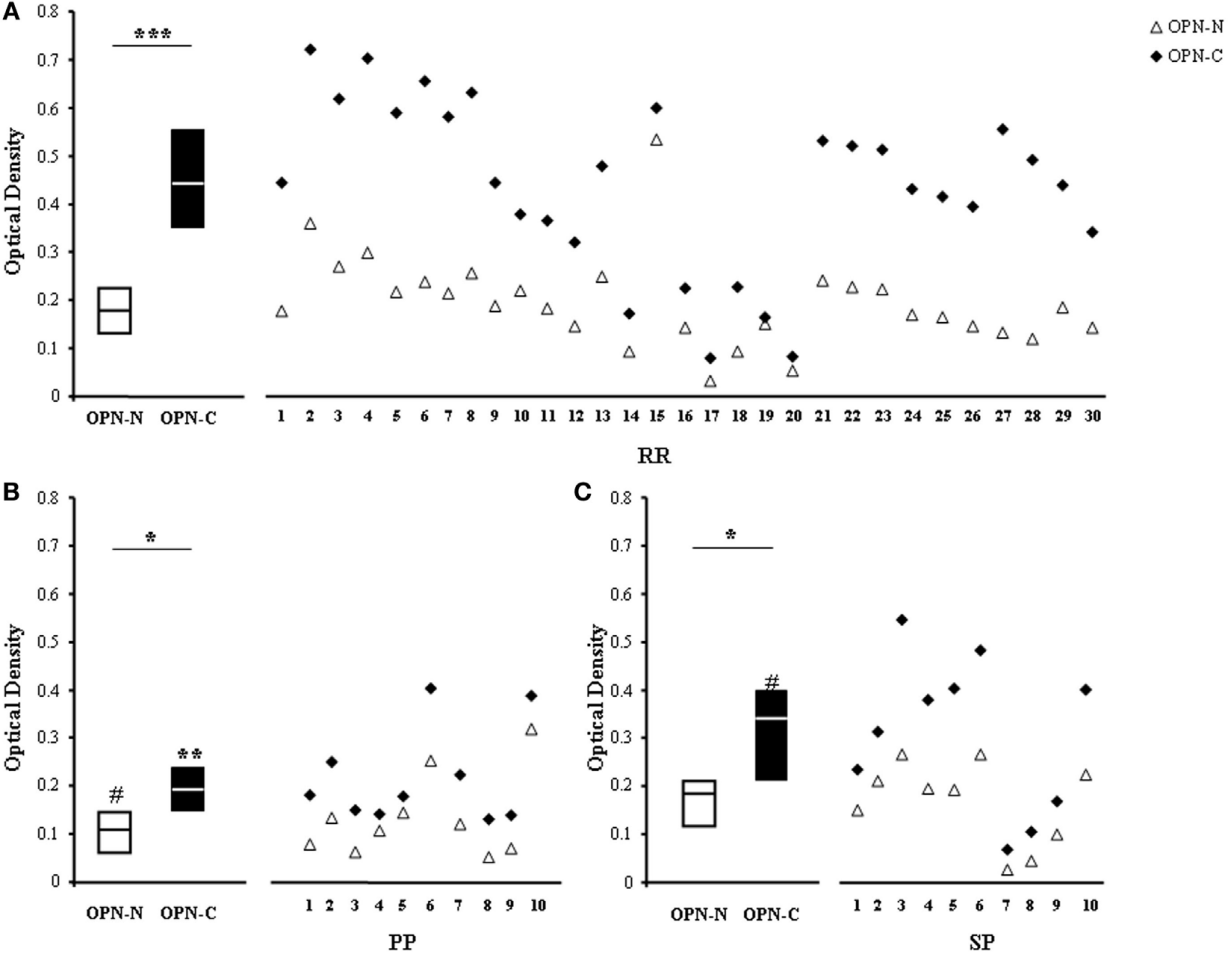
**Autoantibodies (autoAbs) to OPN-N and OPN-C fragments in multiple sclerosis (MS) patients with different clinical forms**. AutoAbs detected by ELISA using either OPN-N (white triangles) or OPN-C (black diamonds) as the capture protein in 30 relapsing–remitting (RR) **(A)**, 10 primary-progressive (PP) **(B)**, and 10 secondary-progressive (SP) **(C)** MS patients. Boxes: interquartile ranges and medians (Wilcoxon test) [*p < 0.05, **p < 0.001, ***p < 0.0001, ^#^*p* < 0.05 vs RR patients (Mann–Whitney *U*-test)].

To map the epitopes recognized by the autoAbs, we selected sera from 30 RR, 10 PP, and 10 SP patients displaying high levels (>75th percentile of the controls) of anti-OPN autoAbs and used the appropriate ELISA to compare their ability to recognize either OPN-C or OPN-N. Figure 4 shows that all sera recognized both OPN-N and OPN-C, but the latter was always more highly recognized than the former. Moreover, the levels of autoAbs against OPN-C were higher in RR than in PP and SP, whereas those against OPN-N were higher in RR and SP than in PP (Figure 4).

### Active Immunization against OPN-C Protects Mice from EAE

To assess the effect of the anti-OPN response *in vivo*, C57BL/6 mice were immunized four times with 10 μg of either mouse OPN-FL or OPN-N or OPN-C cross-linked to OVA. Then, EAE was induced with MOG_35–55_ 1 week after the last immunization.

**Figure 5:**
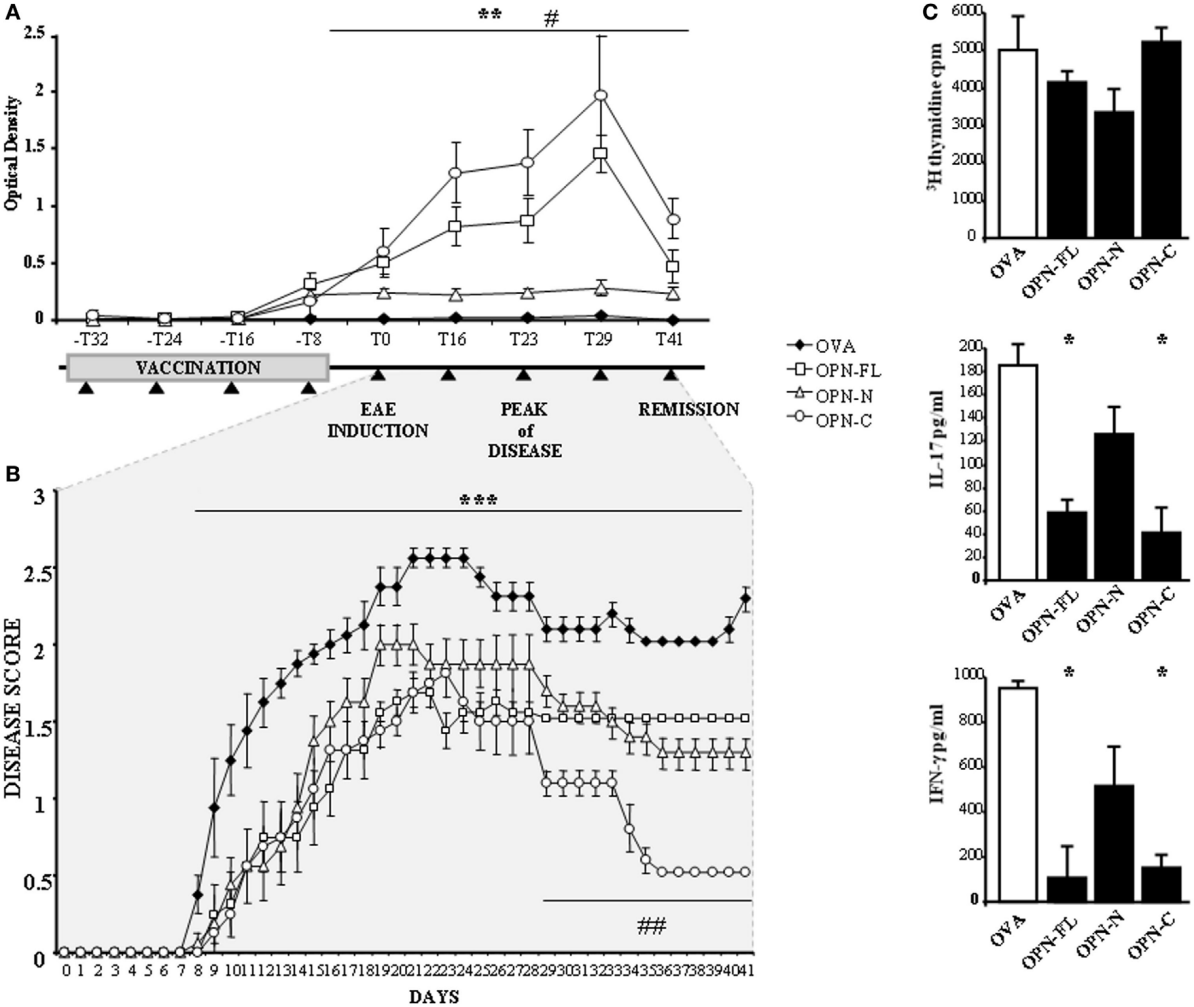
**Vaccination against osteopontin (OPN) induce autoantibodies (autoAbs) to OPN and protects mice from experimental autoimmune encephalomyelitis (EAE)**. **(A)** Anti*-*OPN autoAb levels in vaccinated EAE mice and scheme of the vaccination protocol. Four-week-old female C57BL/6 mice were immunized once a week for 4 weeks with either OPN full-length (OPN-FL) (white squares), OPN-N (white triangles), and OPN-C (white circles) cross-linked to ovalbumin (OVA) or OVA alone as a control (black diamonds). EAE was induced with MOG_35–55_ 1 week after the last immunization. Black triangles indicate blood draws to evaluate OPN autoAbs. The error bars represent the SE for each point [**p < 0.01 OPN-FL and OPN-C vs OVA; ^#^*p* < 0.01 OPN-FL and OPN-C vs OPN-N (Mann–Whitney *U*-test)]. **(B)** Clinical scores of EAE in mice vaccinated as described in panel **(A)**. The error bars represent the SE for each point. A non-parametric ANOVA test was used to compare the clinical scores (***p < 0.001 OPNs vs OVA; ^##^*p* < 0.001 OPN-C vs OVA, OPN-FL, OPN-N). **(C)** *In vitro* response to MOG_35–55_ of spleen lymphocytes from EAE mice vaccinated with OPNs. T cell proliferation, interleukin (IL)-17, and interferon (IFN)-γ secretion were measured in spleen lymphocyte cultures stimulated with MOG_35–55_. Spleen lymphocytes were obtained at day 29 after EAE induction, from mice vaccinated with either OVA or OPN-FL, OPN-N, or OPN-C. Histograms represent the mean ± SE from six mice [*p < 0.05 vs OVA (Mann–Whitney *U*-test)].

The serum levels of anti-OPN autoAbs were evaluated by ELISA using mouse OPN-FL immediately before the first immunization (−T32), at each immunization point (−T24, −T16, −T8), immediately before EAE induction (T0), at three points during the relapse (T16, T23, T29), and in the remission phase (T41). Figure 5A shows that all OPN vaccinations induced anti-OPN autoAbs detectable at the time of the fourth immunization (−T8) but, after 1 week (T0), these autoAbs remained at low levels in the mice vaccinated with OPN-N, while in those vaccinated with either OPN-FL or OPN-C, they increased to a maximum at the peak of disease (T29) and then decreased during the remission phase (T41).

Analysis of the course of EAE showed that the onset of the disease was delayed and its severity reduced by all OPN vaccinations, because the mean clinical score was 1.64 ± 0.14 (mean ± SE) in control mice vaccinated with OVA, compared to 1.04 ± 0.1 (*p* < 0.001), 1.13 ± 0.11 (*p* < 0.001), and 0.81 ± 0.09 (*p* < 0.001) in those vaccinated with OPN-FL, OPN-N, and OPN-C, respectively. Moreover, mice vaccinated with OPN-C displayed faster and more complete remission than those in the other groups (Figure 5B).

To analyze the effect of OPN immunization on the anti-MOG_35–55_ T cells response, spleen lymphocytes were obtained at T29 and cultured for 5 days in the presence of MOG_35–55_. Cell proliferation was then assessed by [^3^H] thymidine uptake, and levels of IFN-γ, IL-4, IL-17A, and IL-10 were evaluated in the culture supernatants by ELISA. Cells from mice vaccinated with either OPN-FL or OPN-C produced lower amounts of IL-17A and IFN-γ than those vaccinated with either OPN-N or OVA (Figure 5C). By contrast, proliferation (Figure 5C) and secretion of IL-10 and IL-4 were not significantly different in the different groups of mice (data not shown).

### Passive Immunization with an Anti-OPN-C mAb Reduces Disability in EAE

**Figure 6:**
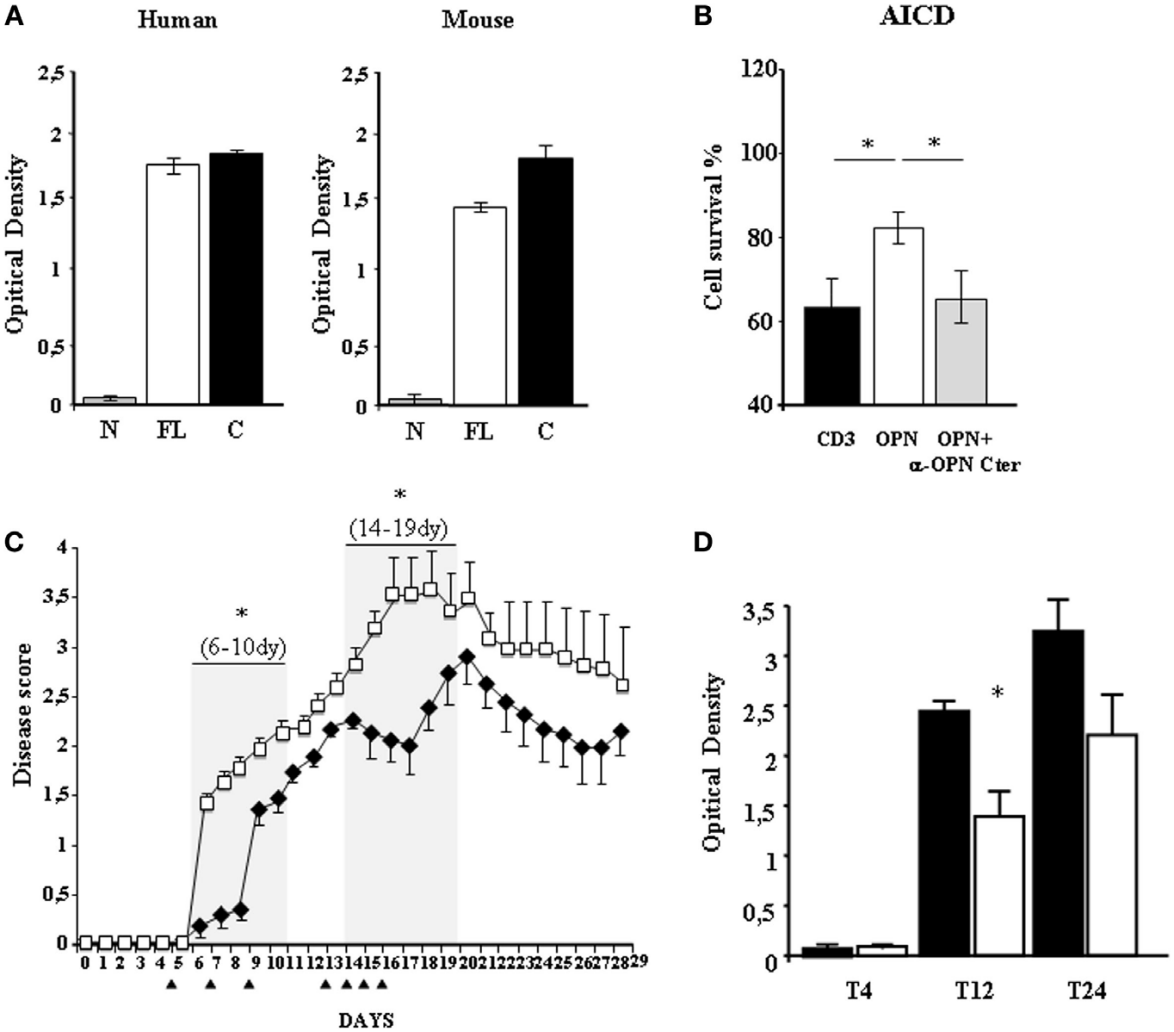
**Passive immunization using an anti-OPN mAb reduces disability in experimental autoimmune encephalomyelitis (EAE)**. **(A)** ELISA testing of the selected mAb, showing its capacity to bind to both human and murine OPN-C, OPN-FL but not to OPN-N. **(B)** The selected mAb neutralizes osteopontin (OPN)-mediated protection from activation-induced cell death (AICD). AICD was induced as described in the legend of Figure 3. The white histogram represents AICD in the presence of recombinant OPN and the gray histogram AICD in the presence of both OPN and the selected mAb, neutralizing it. **(C)** Clinical scores of EAE in mice treated with the selected mAb (black diamonds) or with a control immunoglobulin (Ig)G (white squares). The error bars represent the SE for each point. Black triangles mark the days of treatment and gray boxes the significantly protected days. A non-parametric ANOVA test was used to compare the clinical scores. **(D)** ELISA detecting antibodies against the human determinants of the selected mAb (black) or its isotype control (white), in the serum of treated mice. The anti-mAb response was detectable at T12 and T24 and it was higher in the mice treated with the mAb than in those treated with human IgG [*p < 0.05 (Mann–Whitney *U*-test)].

To assess the *in vivo* effect of the human anti-OPN autoAbs, we produced a human recombinant mAb that was selected according to its capacity to bind human or mouse OPN-C, but not OPN-N (Figure 6A), and to neutralize the human OPN-mediated inhibition of AICD (Figure 6B). The anti-OPN-C mAb or control human IgG were injected i.p. in mice at T5, T7, and T9 after EAE induction. Analysis of the disease scores showed that disability was substantially lower in the mice treated with the mAb than in the control mice in the initial disease phases until T9 (Figure 6C). Subsequently, in the mAb-treated mice, the scores increased abruptly, almost reaching the scores of the control mice from T10 to T13. We then performed another set of four injections of the mAb (or IgG in the control mice) at T13, T14, T15, and T16. In the mice treated with the mAb, the treatment was followed by a decrease of the disease scores until T18, after which the scores again gradually increased and almost reached those of the control mice at T21. Subsequently, both groups of mice developed a similar remission (Figure 6C).

To assess whether the short-lasting effect of the mAb was due to production of antibodies against its human determinants, we searched for these reactive antibodies in the serum of the mice at T4, T12, and T24 using ELISA plates absorbed with the mAb. Results showed that the anti-mAb response was detectable at T12 and T24, and it was higher in the mice treated with the mAb than in those treated with human IgG (Figure 6D; *p* < 0.05).

## Discussion

This study has shown that patients with RR MS display high levels of anti-OPN autoAbs, and these levels are more elevated in remission than in relapse phase. By contrast, these increased levels are not detected in patients with progressive forms of MS, i.e., PP and SP (Figure 1). Moreover, in mouse EAE, vaccination with OPN, boosting production of anti-OPN autoAbs, ameliorates the disease course (Figure 5). These data suggest that production of anti-OPN autoAbs may favor remission in both MS and EAE.

We analyzed correlations between the anti-OPN autoAb levels evaluated at diagnosis and MS clinical course in a prospective cohort of RR patients (24). We found opposite behaviors of OPN autoAbs in patients receiving or not DMTs: a lower disability over 10 years was correlated with high anti-OPN autoAb levels at diagnosis in the former and with low levels in the latter group (Figure 2). Altogether, these findings may have two different, albeit not conflicting, explanations.

First, anti-OPN autoAbs may mark the inflammatory phase of MS. In fact, anti-OPN autoAb levels not only showed an inverse correlation with disease duration but were also higher (0.46 vs 0.26, *p* = 0.0074) in longitudinal RR patients at diagnosis than in cross-sectional RR patients, a portion of whom were tested long after diagnosis, when relapses are less frequent. In cross-sectional RR patients, the autoAb levels were higher during remission than during relapse, thus suggesting that anti-OPN autoAbs are increased by the OPN peak that occurs during relapse. Indeed, high levels were detected in active patients, i.e., both longitudinal RR patients who had subsequently undergone DMTs and those who had not received treatments despite the unfavorable long-term outcome. By contrast, patients with less active disease, i.e., patients not starting DMTs and showing lower MSSS at 10 years, displayed lower antibodies at diagnosis (Figure 2). Overall, these data suggest that high levels of anti-OPN autoAbs at diagnosis may help in identifying active patients requiring DMTs.

**Figure 7:**
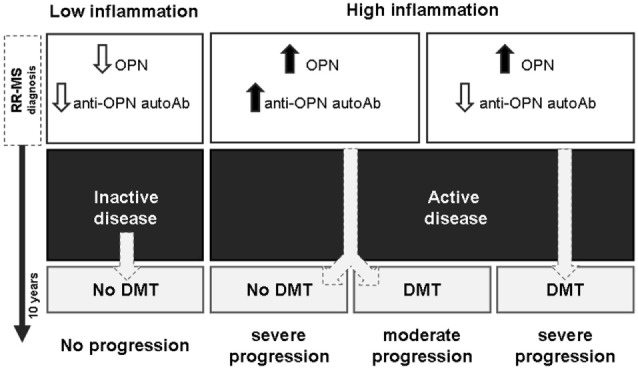
**High anti-OPN autoantibodies (autoAbs) levels in relapsing–remitting (RR) multiple sclerosis (MS) at diagnosis mark active disease and support the efficacy of disease-modifying treatments (DMTs)**. High levels of autoAbs are displayed by patients with active disease including both those who received DMTs and those who did not, despite the subsequent disease progression, which might be associated with the high inflammation and high osteopontin (OPN) levels. However, in the treated patients, the autoAbs may cooperate with DMTs to slow down progression, whereas they are not sufficient to control the disease in the untreated patients.

Second, in patients with active disease, anti-OPN autoAbs may antagonize deleterious activities of OPN involved in MS pathogenesis and cooperate with DMTs to counteract disease progression. These data are in line with those in EAE in which vaccination with OPN, boosting production of anti-OPN autoAbs, ameliorates the disease course and improves remission, as shown also by Steinman et al. (19). Thus, the production of anti-OPN autoAbs may favor remission in both MS and EAE. This model is summarized in Figure 7.

Because the anti-OPN autoAbs were also detected at low levels in the controls, they may be a physiologic response intended to downmodulate the immune response, which is a mechanism that may be shared by other inflammatory cytokines (22). In line with this possibility, we show that the anti-OPN autoAbs are able to neutralize the OPN biologic activity, as detected by their ability to inhibit the OPN-mediated protection on lymphocyte AICD (Figure 3) (17). This neutralization may partly depend on OPN sequestration into immune complexes which may prevent OPN from binding to its cellular receptors and promote OPN removal from the inflamed tissue/bloodstream through the activity of the immune complexes clearing system. However, since the anti-OPN response is polyclonal, it is also possible that some autoAbs have direct blocking effects on the several OPN-binding sites for cellular receptors. Discriminating the role of these binding sites and of molecular and cellular interactions is crucial for designing a specific therapy to target the portion performing the pathogenic function while preserving the physiologic activity of the others.

The notion of the protective effect of autoAbs in autoimmune diseases is also supported by clinical experience with B-cell-depleting treatments. While use of anti-CD20 antibodies, such as rituximab and ocrelizumab, is considered an important therapeutic strategy in MS (30), two randomized controlled clinical trials with atacicept in MS and optic neuritis were discontinued for significant disease worsening in the treatment compared to the placebo arms (31, 32). A possible explanation for this discrepancy between the two B-cell-depleting treatments is that anti-CD20 antibodies relatively spear plasma cells, thus allowing the production of protective antibodies. On the contrary, atacicept significantly reduces serum Ig concentrations, mature B cells but also plasma cells (33).

A major objective at MS diagnosis is to act at the early inflammatory stage, delaying disease progression and development of disability. Patients with RR MS may benefit from DMTs showing different degrees of both efficacy and side effects. Personalized treatment is a key challenge in decision-making with regard to MS because of the shortage of reliable markers of individual disease prognosis. Therefore, OPN and anti-OPN autoAbs might be valuable tools in this scenario. OPN levels have been analyzed extensively in MS, as biomarkers of disease activity and DMTs effectiveness (34, 35), and displaying correlations with clinical outcome. These findings have been useful to depict the immunopathological role of OPN in MS, but not to evaluate MS prognosis because of the wide variability of the OPN levels in different clinical conditions and experimental settings (36, 37). These inconsistencies may be related to the numerous clinical conditions that may influence OPN levels, the difficulty of detecting the different OPN forms (depending on glycosylation, phosphorylation, and proteolytic cleavage), and the variable amount of OPN included in the immune complexes with its autoAbs. Similarly, also detection of anti-OPN autoAbs may be misleading because they may be part of the immune complexes and vary in their ability to neutralize the multiple functions of OPN. It is possible that the best approach would be the parallel evaluation of both free and immune complexes-bound OPN and anti-OPN autoAbs.

Intriguingly, the anti-OPN response recognized OPN-C better than OPN-N in all patients, which may mark both quantitative (i.e., different amounts) and qualitative (i.e., different affinities) differences of the autoAbs produced against the two fragments (Figure 4). The focus on OPN-C was further noted by the EAE experiments because vaccination with OPN-C resulted in the greatest induction of anti-OPN autoAbs, ameliorating disease progression, particularly in terms of inducing disease remission and decreasing the autoantigen-driven production of IFN-γ and IL-17 (Figure 5). Moreover, passive immunization with the human anti-OPN-C recombinant antibody ameliorated the disease course (Figure 6). These data identified a role for the CD44-binding site displayed by OPN-C, which is intriguing because CD44 is involved in EAE by favoring the homing and survival of the autoimmune T cells, and by increasing IL*-*17A and IFN-γ production and decreasing IL-10 production (34–42). Moreover, data in the literature show that OPN stimulates IL*-*17A and IFN-γ production and inhibits IL-10 production in EAE and MS (15).

The critical role of OPN-C is surprising because the presence of the binding sites for α4β1 would instead direct the attention to OPN-N because α4β1 is involved in the CNS homing of T cells and is the target of the anti-MS drug natalizumab. However, it is noteworthy that our data indicate that OPN-N also plays a role in EAE, because vaccination with OPN-N ameliorated disease progression. Moreover, when we analyzed the autoAbs to OPN-C and OPN-N in the longitudinal group of RR-MS patients at diagnosis, we could not confirm the clinical correlations detected on the total anti-OPN autoAbs, which highlights the importance of the global response to OPN (data not shown).

In the EAE experiments, we used a prophylactic vaccination protocol in which immunization was performed before EAE induction. This procedure would be of limited benefit in humans, who would instead benefit from a therapeutic vaccination performed after the onset of disease. However, even a therapeutic vaccination would be problematic in humans because of the concern about inducing an uncontrollable anti-OPN response. By contrast, a possible approach would be to use anti-OPN-neutralizing antibodies, since we show that they can ameliorate EAE disability when administered in different phases of the disease. In our model, the effect was short-lasting, but this was probably due to the high anti-drug response elicited by the human mAb used in these experiments.

Osteopontin has pleiotropic activities in the immune response because it acts as a chemoattractant for inflammatory cells, supports differentiation of proinflammatory T cells and antibody production by B cells, and increases survival of activated lymphocytes and inflammatory cells (4, 39, 40, 43, 44). Discriminating the role of each fragment in these functions and in MS pathogenesis is crucial for designing a specific therapy to counteract only the most pathogenic fragment and function while preserving the physiologic activity of the others. This work is a proof of concept that drugs targeting OPN-C may be proposed for MS therapy.

We have shown that anti-OPN autoAbs are found at high levels in RR-MS patients during the remission, and that they influence MS evolution and prognosis in association with DMTs. Novel strategies boosting their levels, such as vaccination or passive immunization, may be proposed as a future strategy in personalized MS therapy.

## Author Contributions

NC, DR, GC, EO, CG, EB, and CD performed the experiments and analyzed the data. DS performed the phage display screening experiments; MS, FM-B, MC, AB, LA, ML, CC, and DV provided the patient samples and clinical data; TC performed the statistical analysis; CC, UD, and AC designed the study and wrote the manuscript.

## Conflict of Interest Statement

The authors declare that the research was conducted in the absence of any commercial or financial relationships that could be construed as a potential conflict of interest.
